# Burns in pregnancy: a case report from Buea Regional Hospital, Cameroon

**Published:** 2009-12-22

**Authors:** John Ahmadou Mokube, Vincent Siysi Verla, Victor Njie Mbome, Aimié Thierry Bitang

**Affiliations:** 1 Faculty of Health Sciences, University of Buea, Cameroon;; 2 Buea Regional Hospital, Cameroon

**Keywords:** Burns, pregnancy, multidisciplinary approach, prognosis

## Abstract

**Background:**

To the best of our knowledge similar cases of severe burns in pregnancy have not been published in Cameroon; indicating the rarity of this devastating condition and therefore the dilemma that practitioners may be confronted with in its management. This report is to help the Physician understand the factors that should determine his/her management decisions by reviewing the limited literature of burns in pregnancy. A multidisciplinary approach by a team of Obstetricians, Anesthetist/Intensive care Physicians, Pediatricians and Surgeons is indispensible. Adequate resuscitation, fight against sepsis, the gestational age and the severity of the burn will determine the outcome or prognosis.

## Background

We have never encountered or been confronted with a case of burns in pregnancy during the 20–30 years of medical practice, nor have we come across text books treating this severe trauma of burns in pregnancy. Furthermore, this has not been taught as a lesson in Obstetrics and Intensive Care. Therefore, it is of paramount importance to report this case in order to highlight the dilemma or the difficulties faced by practitioners in managing severe burns and their effects in pregnancy. We also want to review the limited medical literature related to handling of such cases, with the goal of reducing maternal and fetal morbidity and mortality. Our case is that of severe burns in an obese woman who was admitted in our Hospital on 4 March 2009.

## Case report

A B.Y., age 26yrs, G _3_ P _1011_ , third trimester (at term) with the estimated date of confinement on 09/03/2009, suffered a second degree, 28% burned surface area on 04/03/2009. This was due to flames from a domestic gas explosion (accident), following leakage of gas in a poorly ventilated room. The husband and a 4-year old son also sustained 2 ^nd^ degree thermal injuries of 12 % and 7 %, respectively.

On physical examination, she was obese, and in severe pains with the following parameters; blood pressure=90/45mmHg, pulse rate=120bpm, T=36.6 degrees Celsius, respiratory rate=24 breaths per minute. According to her antenatal records, she weighed 96kg before the pregnancy, with a height of 163cm and hence Body mass index (BMI) of about 36kg/m ^2^ . She weighed 107 kg, two weeks before the burn injury.

**Figure 1:**
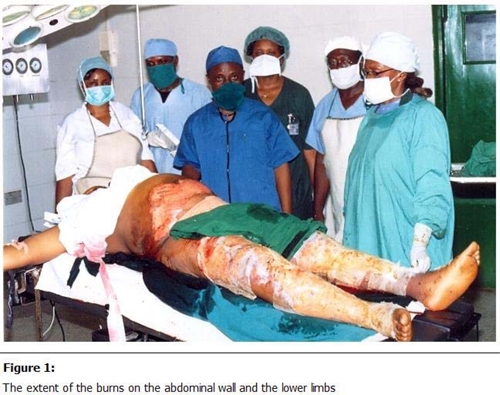
The extent of the burns on the abdominal wall and the lower limbs.

Her skin was burned on the abdomen just above the Pfannenstiel incision line and above the umbilicus and across to the flanks. Both lower limbs were extensively burned, sparing the perineal region and the feet. Total burn surface area was estimated at 28% ( figure 1 ). The foetal heart sounds were heard and the rate was 140 beat per minute. Meanwhile foetal movements were felt by the mother. The presentation was cephalic. She was administered adequate fluids intravenously according to the Parkland Hospital formula and asked to drink as required (pro re nata). She also received analgesics (Tramadol hydrochloride 100mg 12-hourly, subcutaneously and Paracetamol 1000 mg 8-hourly infusion). She was also administered sulbutamol (tocolytic) and phloroglucinol a spasmolytic agent to control uterine contractions. Ceftriazone 1g was given 12-hourly intravenous to prevent sepsis. Meanwhile ranitidine 50 mg was given 8-hourly IV to guard against gastric perforation. We nursed her in the semi-upright position with the body tilted to the left. Preoperatively her bleeding time (2 min 30 seconds) and the clotting time (7 min 30 seconds) were within normal ranges; the haemoglobin and haematocrit were 13.5g/dl and 33.5% respectively.

**Figure 2:**
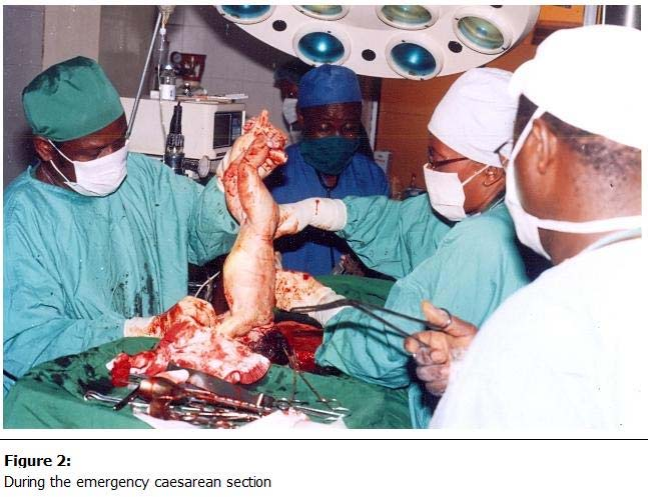
During the emergency caesarean section.

**Figure 3:**
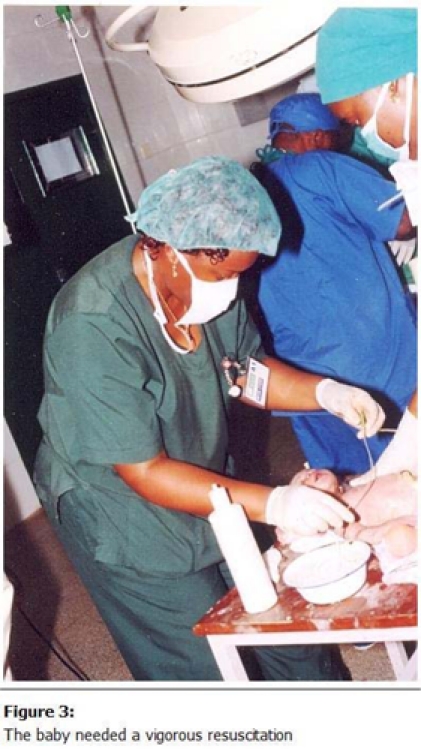
The baby needed a vigorous resuscitation.

On the 09 of March 2009 which was the expected day of confinement, we were still contemplating on the most appropriate mode of delivery when she complained of reduced foetal movements (activities). A quick assessment confirmed foetal distress. Then we decided to do an emergency caesarian section ( figure 2 ) under a balanced general anaesthesia with ketamine after adequate oxygenation for a few hours. The female baby needed a vigorous resuscitation ( figure 3 ) that we had anticipated. Apgar score was 4/10 in the 1 ^st^ minute, 6/10 in 5 ^th^ minute and 8/10 in the 10 ^th^ minute. The amniotic fluid was yellowish. The mother was put on triple antibiotics (ceftriazone, metronidazole and gentamicin) in the postoperative period and the baby on Ampicillin and gentamicin. The baby started breast feeding immediately. She developed jaundice on day two that subsided in a few days.

The mother continued medical and surgical treatment and was discharged after four weeks. The husband and son had earlier been discharged.

## Discussion

According to Napoli B et al, texts in Obstetrics do not deal with burns in pregnancy nor is the topic considered in books devoted to the treatment of burns [1]. According to Ghotbi et al, there are no written protocols as how to manage burns in pregnancy but the most important decision to be made is whether the pregnancy should be terminated or not [2]. In our case the pregnancy was at term, but we were faced by many dilemmas; adding injury (pains of labour) to the trauma by allowing the woman to go into labour? What if foetal distress is augmented during the several hours of labour? Are we not increasing the risk of foetal infection during vaginal delivery from the burns site or laps? What of difficulty in opening her legs for vaginal delivery? Can she withstand the extra pains? Is it possible to conduct a vaginal delivery under these conditions? According to R.N Mathews, vaginal delivery has always been proved possible, even in the presence of perineal burns but caesarian section should be considered and may be preferred [3]. Burn injury during pregnancy is a serious problem that needs close co-operation or a multidisciplinary approach [4]. It needed the combined efforts of an obstetrician/gynecologist and anesthetist/intensive care physician and others to manage the case reported. This required early and adequate fluid resuscitation, avoiding hypovolaemic shock, a cause of decreased uterine blood flow leading to foetal hypoxia, and the use of limited choice of antibiotics to avoid septicemia, and analgesic [1].

It is said that vaginal delivery is possible, even in the presence of perineal burns but that caesarean section should be considered and may be preferred. In our case, after considering or examining the questions above, we opted for a c/s. The gestational period is one of the factors that determine the obstetric procedure (no intervention, protection of pregnancy by the use of tocolytics, induction and/or acceleration of labour [1]. In our case, third trimester we did a c/s, after having used tocolytics to delay delivery because of the high risk of infection.

A 28% (table of Lund and Browder ) burned surface area, second degree burns in a pregnant obese lady is classified under severe burns. She also had a score of 5 points on the abbreviated burn severity(ABS) index of Tobiasen et al giving her a survival probability of 0.98 [5]. This is in the developed world where we have the best medical conditions.

It is good to nurse the parturient in the semi- upright position and also to administer 02 in order to avoid hypoxia [1]. In our case the patient was nursed in the semi upright position, tilted to the left. She was severely obese; a factor of modification (decrease) of pulmonary volumes and capacities [6–7] and this is exacerbated by pulmonary changes associated with pregnancy [8–9]. We basically did a prompt and aggressive fluid therapy, vigorous antibiotic cover for sepsis, oxygenation and delivery of the foetus (by c/s) because the pregnancy was in third trimester and at term [10].

## Conclusion

A severe burn in pregnancy is a complicated and devastating condition. Management requires a multidisciplinary approach with close monitoring of maternal and foetal well-being. Delivery should be done as early as possible especially at or near term.
